# Maternal Vulnerability Indicators Derived from Free-Text Clinical Documentation in Adolescent Pregnancy

**DOI:** 10.3390/medicina62081598

**Published:** 2026-08-20

**Authors:** Florin Mihai Sandor, Cris Virgiliu Precup, Cristian George Furau, Antoine Edu, Mihnea-Dan Edu, Ciprian Coroleuca, Roxana Furau, Ioana Roșca, Radu Nicolae Mateescu

**Affiliations:** 1 Multidisciplinary Doctoral School, “Vasile Goldis” Western University of Arad, 310414 Arad, Romania; sandor.florin@uvvg.ro (F.M.S.); furau.cristian@uvvg.ro (C.G.F.); 2 Department of Life Sciences, “Vasile Goldis” Western University of Arad, 310414 Arad, Romania; precup.cirs@uvvg.ro; 3 Department 13—Obstetrics and Gynecology, Faculty of Medicine, “Carol Davila” University of Medicine and Pharmacy, 020021 Bucharest, Romania; antoine.edu@umfcd.ro (A.E.); ciprian.coroleuca@umfcd.ro (C.C.); drmateescuradu@gmail.com (R.N.M.); 4 Nicolae Malaxa Clinical Hospital, 022441 Bucharest, Romania; 5 Panait Sârbu Clinical Hospital of Obstetrics and Gynecology, 060251 Bucharest, Romania; ioana.rosca@umfcd.ro; 6 Department of General Medicine, Faculty of Medicine, “Vasile Goldis” Western University of Arad, 310414 Arad, Romania; 7 Faculty of Midwifery and Nursery, “Carol Davila” University of Medicine and Pharmacy, 050474 Bucharest, Romania

**Keywords:** adolescent pregnancy, maternal vulnerability, narrative clinical documentation, routinely collected health data, prenatal care deficiency, low birth weight, perinatal epidemiology

## Abstract

*Background and Objectives*: Adolescent pregnancy is associated with biological, behavioral, and healthcare-related vulnerabilities that are incompletely captured by structured datasets. This study aimed to characterize selected maternal vulnerability indicators derived from narrative clinical documentation and to examine their exploratory associations with documented prenatal care deficiency and neonatal outcomes. *Materials and Methods*: We conducted a retrospective cohort study including 759 singleton adolescent pregnancies managed at a Romanian secondary obstetric center between 2020 and 2024. Narrative clinical observations were reviewed to extract maternal vulnerability indicators, including smoking, anemia, infection, hypertensive disorders, obesity, Rh incompatibility, and documented prenatal care deficiency. Associations between extracted variables, documented prenatal care deficiency, and neonatal outcomes were evaluated using contingency analyses, Kendall’s tau-b correlations, and multivariable logistic regression models. *Results*: Among pregnancies with available narrative observations, documented prenatal care deficiency was identified in 612/692 (88.4%), maternal anemia in 207/692 (29.9%), and smoking during pregnancy in 186/692 (26.9%). Validation performance differed across categories, with a sensitivity of 57.1% (95% CI 18.4–90.1%) for hypertensive disorders and wide confidence intervals for rare categories. Correlations between extracted variables were weak, indicating limited clustering of maternal risk factors. Hypertensive disorders (aOR 0.31, 95% CI 0.10–0.95, *p* = 0.038) and maternal obesity (aOR 0.39, 95% CI 0.16–0.98, *p* = 0.044) were associated with lower odds of documented prenatal care deficiency. Significant neonatal associations were observed primarily for low birth weight. Hypertensive disorders were independently associated with increased odds of low birth weight (aOR 3.56, 95% CI 1.33–9.54, *p* = 0.011), whereas documented maternal anemia was associated with lower observed odds of LBW (aOR 0.45, 95% CI 0.24–0.82, *p* = 0.009). No significant associations were identified for preterm birth or Apgar score < 7 at 5 min. *Conclusions*: Narrative clinical documentation allowed the identification of heterogeneous maternal vulnerability indicators that showed weak interrelationships and selective associations with prenatal surveillance patterns and low birth weight. These exploratory findings should be interpreted in the context of healthcare engagement and documentation practices.

## 1. Introduction

Adolescent pregnancy remains a major public health challenge worldwide despite substantial reductions in adolescent fertility rates observed over recent decades. Pregnancies occurring before the age of 18 years continue to be associated with increased risks of maternal complications, adverse neonatal outcomes, and long-term social vulnerability, particularly in populations characterized by limited healthcare access and socioeconomic disadvantage [1,2]. Large population-based studies have consistently demonstrated higher rates of low birth weight, preterm birth, fetal growth restriction, and neonatal morbidity among adolescent mothers compared to adult women [3]. Although biological immaturity may contribute to some of these outcomes, growing evidence suggests that the adverse prognosis associated with adolescent pregnancy is strongly influenced by broader contextual determinants, including poverty, documented prenatal care deficiency, nutritional deficiencies, smoking, unstable social environments, and reduced healthcare engagement [4].

The concept of maternal vulnerability has therefore gained increasing relevance in perinatal epidemiology. Rather than representing a single clinical condition, maternal vulnerability encompasses the interaction between biological, behavioral, psychological, and social risk factors that may compromise pregnancy monitoring and neonatal health [5]. In adolescent populations, these dimensions frequently coexist and may remain insufficiently captured by conventional structured datasets. Previous research has shown that adolescent mothers are more likely to experience delayed antenatal booking, inconsistent pregnancy surveillance, anemia, hypertensive disorders, substance exposure, psychosocial stressors, and barriers to continuity of care [6,7,8]. However, many of these vulnerabilities are documented descriptively in clinical narratives rather than systematically encoded in standardized electronic variables [9]. As a result, studies based on routine clinical records often rely on selected vulnerability-related indicators that can be consistently identified and analyzed within the available documentation [9,10].

The increasing implementation of electronic health records has generated growing interest in the epidemiological use of unstructured clinical data. Free-text clinical documentation may contain information that is incompletely represented in structured registries [11,12].

Natural language processing and narrative data extraction approaches have been increasingly used to identify clinical phenotypes, comorbidities, and healthcare patterns across multiple medical disciplines [13]. These approaches range from automated natural language processing techniques to rule-based extraction strategies that transform narrative clinical observations into structured variables suitable for epidemiological analysis [9,13]. Rule-based approaches remain particularly useful when clinically relevant information is documented using recurrent and explicit terminology.

In obstetrics, routine narrative documentation may provide additional insight into maternal smoking, inadequate prenatal monitoring, obesity, infection, anemia, or hypertensive disorders, particularly in healthcare systems where structured coding remains incomplete or inconsistently applied.

At the same time, interpretation of free-text-derived variables requires considerable caution. Clinical narratives do not exclusively reflect disease burden; they may also encode differences in healthcare access, clinician awareness, diagnostic intensity, and documentation practices [9]. Conditions such as hypertension or obesity may be more frequently identified among women receiving adequate prenatal care simply because these pregnancies undergo more intensive surveillance. Conversely, the absence of documented pathology in poorly monitored pregnancies may reflect underdiagnosis rather than a true absence of risk. Consequently, variables extracted from routine free-text observations may simultaneously represent biological vulnerability and patterns of interaction with the healthcare system [10]. This phenomenon highlights a critical systemic confounder in electronic health records, where the non-random frequency of medical encounters generates an informed presence bias [10,14]. In the context of vulnerable populations, unstructured clinical notes do not merely record objective clinical events; instead, they capture an intersection of patient biology, health-seeking behaviors, and documentation practices that can mask underlying risks through underdiagnosis [14].

This distinction may be particularly important when evaluating neonatal outcomes. Low birth weight is strongly associated with maternal nutritional status, placental dysfunction, hypertensive disease, smoking, and documented prenatal care deficiency [15,16]. Preterm birth similarly reflects a multifactorial process involving inflammatory, obstetric, behavioral, and socioeconomic determinants [17]. In contrast, low Apgar score may depend more directly on intrapartum complications, gestational maturity, and immediate neonatal adaptation than on chronic maternal conditions documented during pregnancy [18]. Assessing these outcomes separately may therefore clarify whether free-text-derived maternal variables capture generalized perinatal vulnerability or outcome-specific risk patterns.

Despite the expanding use of electronic health record research, relatively few studies have examined the use and interpretative limitations of routine narrative documentation in adolescent obstetric populations. Most available studies rely predominantly on structured variables, administrative coding, or predefined clinical diagnoses, potentially overlooking contextual information embedded within narrative observations. Furthermore, the relationship between free-text-derived maternal risk factors and neonatal outcomes remains insufficiently understood, particularly in vulnerable adolescent cohorts.

This study aimed to characterize maternal vulnerability indicators identified from routine free-text clinical documentation in adolescent pregnancies and to evaluate their exploratory associations with documented prenatal care deficiency and neonatal outcomes. Specifically, we analyzed smoking, anemia, infection, hypertensive disorders, obesity, Rh incompatibility, and documented prenatal care deficiency among adolescent mothers younger than 18 years and examined their relationships with low birth weight, preterm birth, and low Apgar score. By focusing on routinely recorded narrative clinical observations, the study sought to describe selected maternal vulnerability indicators captured in the available free-text documentation and to examine the interpretative challenges associated with their use in retrospective epidemiological research.

## 2. Materials and Methods

### 2.1. Study Design and Setting

This retrospective cohort study included adolescent mothers younger than 18 years who delivered at the Obstetrics and Gynecology Clinic of the Arad County Emergency Clinical Hospital, Romania, between 1 January 2020 and 31 December 2024. The study was conducted using routinely collected institutional birth-registry data and retrospectively abstracted hospital documentation, including general clinical observation charts and medical letters/discharge summaries. The reporting of the study was informed by the RECORD (Reporting of Studies Conducted Using Observational Routinely Collected Health Data) recommendations for studies based on routinely collected health data [19].

All deliveries occurring during the study period were screened for eligibility. A total of 763 adolescent deliveries were initially identified. Multiple pregnancies (n = 3) were excluded because of their distinct obstetric and neonatal risk profile. One additional case was excluded because the record lacked sufficient clinical information to determine pregnancy characteristics and allow variable extraction. The final cohort consisted of 759 singleton adolescent pregnancies.

**Figure 1:**
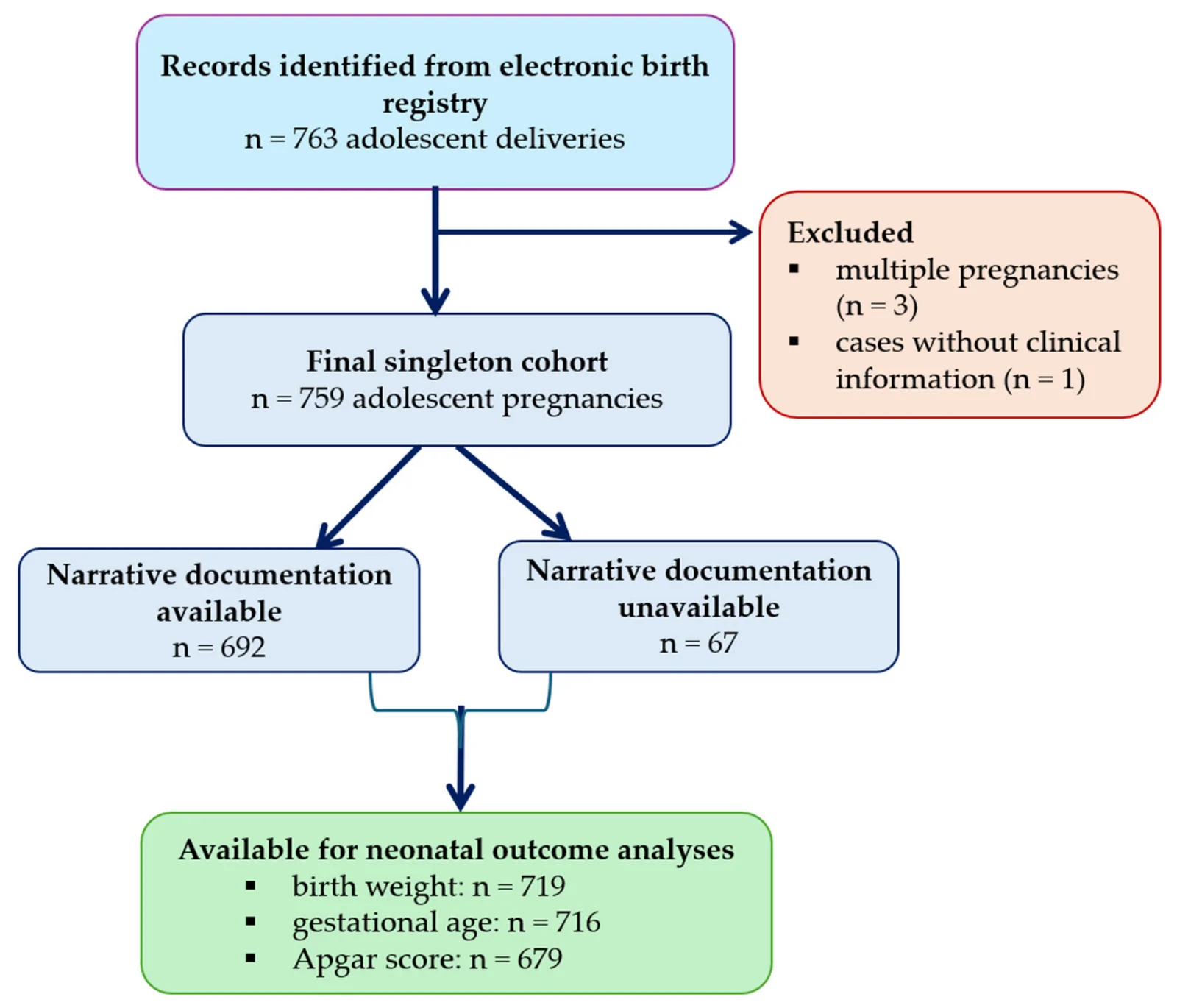
Flow diagram of cohort selection and data availability for free-text clinical observations and neonatal outcomes.

The study flowchart and availability of free-text and neonatal outcome data are presented in Figure 1.

Narrative clinical observations were available for 692 pregnancies and constituted the analytical sample for the free-text component of the study. Because the extraction of vulnerability-related indicators required the presence of narrative documentation, pregnancies without free-text observations (n = 67) could not be included in these analyses. To assess the potential for selection bias, pregnancies with and without narrative documentation were subsequently compared with respect to major maternal and neonatal characteristics.

### 2.2. Data Sources and Clinical Variables

The present study was conducted using the same institutional cohort of adolescent pregnancies previously examined in two complementary analyses: an age-stratified study focused on maternal age and obstetric and intrapartum outcomes, and a parity-based study focused on neonatal outcomes [20,21]. Maternal demographic, obstetric, and neonatal data were obtained from routinely collected institutional hospital records. Structured variables included maternal age, residential environment (urban/rural), gestational age at delivery, birth weight, and Apgar score at 5 min. Neonatal outcomes were analyzed as binary variables. Low birth weight (LBW) was defined as birth weight below 2500 g. Preterm birth was defined as delivery before 37 completed weeks of gestation. Low neonatal adaptation was defined as an Apgar score below 7 at 5 min. These outcomes were selected because they represent clinically relevant indicators of neonatal morbidity and are commonly used in perinatal epidemiological research.

In addition to the structured obstetric and neonatal variables, narrative clinical information was retrospectively abstracted from the general clinical observation charts and the medical letters/discharge summaries archived by the maternity hospital. These sources covered the hospitalization associated with delivery and included clinically relevant maternal and pregnancy-related information documented during the admission. The study did not include longitudinal outpatient prenatal-clinic records or a complete chronological review of all individual inpatient progress notes. Final diagnosis codes were not available as structured variables in the analytic extract and were therefore not analyzed separately.

The structured analytic extract contained no structured counterparts for the seven narrative-derived vulnerability indicators. Consequently, the study could not quantify cases identified exclusively through narrative review, assess agreement with structured diagnoses, estimate incremental informational or prognostic value, or evaluate changes in patient classification after the addition of narrative data.

### 2.3. Free-Text Harmonization and Rule-Based Extraction

The abstracted narrative information was centralized and processed in Microsoft Excel. Because the analysis relied on routine clinical documentation, only conditions explicitly recorded in the reviewed source documents could be coded. Absence of documentation was therefore interpreted as absence of documented evidence within the available records, rather than as definitive absence of the corresponding clinical condition. Prior to variable extraction, free-text entries underwent manual preprocessing to reduce inconsistencies related to punctuation, spelling variations, abbreviations, synonymous terminology, and the use of Romanian diacritics. Clinically equivalent expressions were standardized using a predefined harmonization dictionary. The harmonization dictionary was initially developed by a senior obstetrician with extensive clinical experience in the management of adolescent pregnancies at the study institution and familiarity with the terminology routinely used in institutional clinical documentation. Narrative observations were reviewed iteratively to identify recurrent synonymous expressions, abbreviations, spelling variants, and alternative clinical formulations referring to the same underlying clinical concept. These expressions were consolidated into predefined standardized categories, which were progressively refined until no additional clinically meaningful variants were identified.

**Figure 2:**
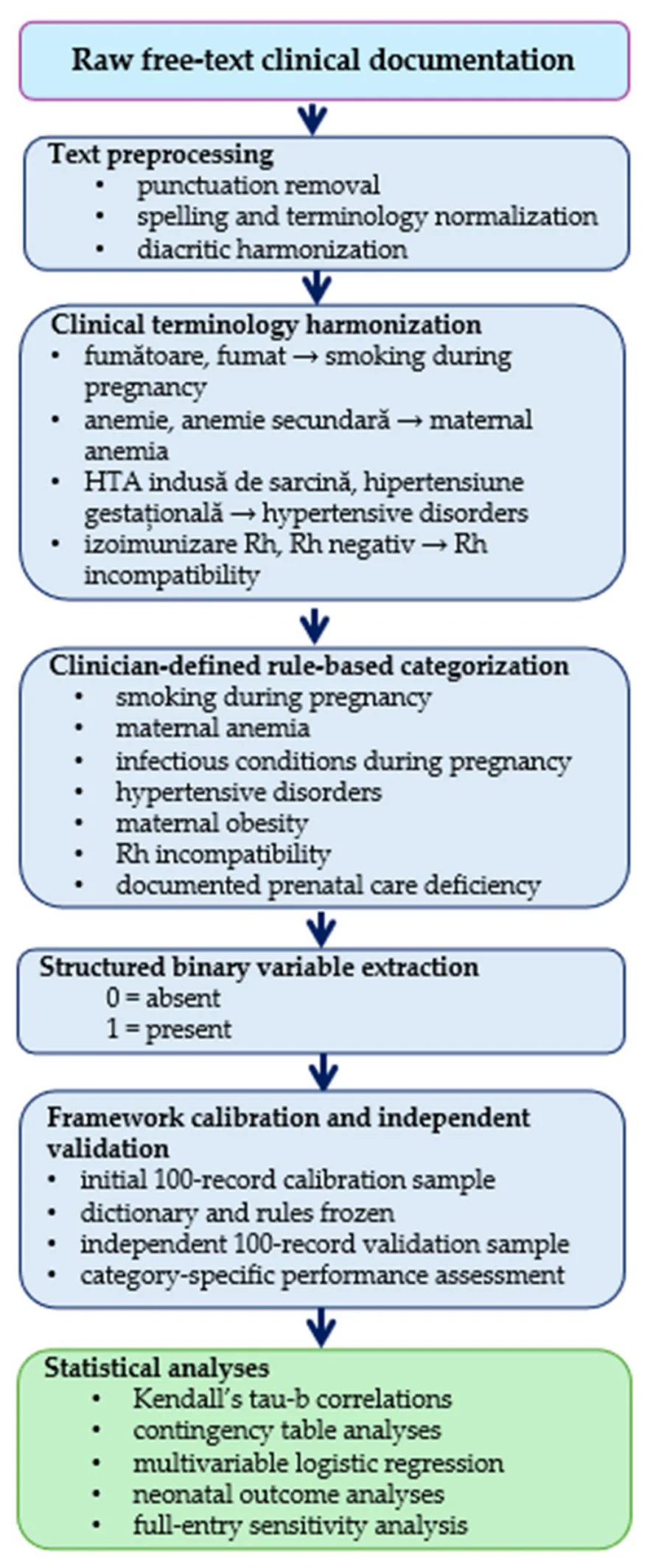
Workflow for preprocessing, terminology harmonization, clinician-defined rule-based categorization, binary extraction, and validation of maternal vulnerability indicators derived from routine narrative obstetric documentation.

Following text standardization, a rule-based clinician-defined coding framework was applied. The objective of the framework was not automated phenotype discovery but the reproducible identification of predefined maternal vulnerability indicators considered clinically relevant in adolescent pregnancy. Recurrent synonymous expressions were mapped to predefined maternal vulnerability categories. The complete workflow used for preprocessing, harmonization, categorization, and binary variable generation is presented in Figure 2.

An initial random sample of 100 narrative records was used to evaluate the implementation of the predefined extraction framework. This calibration review identified two mapping errors involving expressions already covered by the dictionary: the Romanian term “picnică”, corresponding to pyknic habitus within the maternal obesity category, and the abbreviation “IACRS”, corresponding to an acute upper respiratory tract infection within the infectious-conditions category. No new vulnerability categories or operational definitions were introduced. After correction of these implementation errors, the harmonization dictionary, operational definitions, and extraction rules were finalized and frozen.

For independent internal validation, a second random sample of 100 records was selected without replacement from records not included in the initial calibration sample. Selection was performed using a computer-generated random sequence with a fixed seed of 20260728. The frozen rule-based extraction framework was applied to these records without further modification. A second clinician, blinded to the rule-based classifications, independently reviewed the original narrative documentation and assigned binary classifications for each of the seven predefined maternal vulnerability indicators.

After completion of the clinician and rule-based classifications, discordant classification pairs were reviewed against the original narratives. Three clinician entries, two in the infection category and one in the obesity category, were identified after the classifications had been compared and were confirmed by the clinician as category data-entry errors. These entries were corrected for the primary validation analysis. The remaining discrepancies were retained as validation disagreements, and the frozen dictionary, operational definitions, and extraction rules were not modified. Because the three errors were identified after comparison of the classifications, a sensitivity analysis was also performed using the original, uncorrected clinician entries.

The independent clinician classifications were treated as the reference standard for the validation analysis. For each predefined indicator, a complete 2 × 2 classification table was constructed, and sensitivity, specificity, positive predictive value, negative predictive value, percentage agreement, and Cohen’s kappa were calculated. Exact binomial 95% confidence intervals were reported for sensitivity, specificity, positive predictive value, and negative predictive value. Because the prevalence of some indicators was low in the validation sample, category-specific estimates were interpreted with particular caution. Overall percentage agreement and a descriptive pooled Cohen’s kappa were calculated only as supplementary summary measures and were not used in place of category-specific performance estimates.

Following harmonization, narrative expressions were grouped into predefined maternal vulnerability categories according to a clinician-defined harmonization dictionary. The standardized expressions included in each category are summarized in Table 1, whereas the complete harmonization dictionary is available in Supplementary Table S1.

Only predefined maternal vulnerability indicators were retained for statistical analysis. Variables selected a priori included:

- Smoking during pregnancy;
- Maternal anemia;
- Infectious conditions during pregnancy;
- Hypertensive disorders;
- Maternal obesity;
- Rh incompatibility;
- Documented prenatal care deficiency.

The selected indicators were chosen a priori based on their clinical relevance and the availability of explicit documentation within routine obstetric records. These variables were intended to represent selected vulnerability-related characteristics identifiable within narrative clinical documentation and were not designed to capture the full multidimensional construct of maternal vulnerability.

Documented prenatal care deficiency was defined as the presence of narrative clinical documentation explicitly indicating absent, insufficient, delayed, or irregular antenatal monitoring. This variable reflected clinician-documented deficiencies in prenatal surveillance rather than a guideline-based assessment of antenatal care adequacy. Because expressions such as “insufficiently investigated pregnancy”, “uninvestigated pregnancy”, and “non-dispensarized pregnancy” shared the common clinical meaning of inadequate antenatal surveillance, they were grouped into a single category representing documented prenatal care deficiency.

The predefined categories were subsequently transformed into binary variables indicating the presence (1) or absence (0) of each maternal vulnerability indicator. Clinical observations that did not correspond to the predefined extraction framework were not included in the final analyses. Cases lacking narrative clinical observations were treated as missing for narrative-derived variables and excluded from analyses involving those specific indicators. Because the reasons for missing narrative observations could not be directly determined from the available records, the missing data mechanism was considered potentially non-random.

### 2.4. Statistical Analysis

Statistical analyses were performed using JASP statistical software (version 0.19.3). Categorical variables were summarized as frequencies and percentages, while continuous variables were described using means and standard deviations or medians and interquartile ranges, as appropriate according to distribution characteristics.

Associations between free-text-derived maternal vulnerability indicators, prenatal care adequacy, and neonatal outcomes were initially evaluated using contingency table analysis and Pearson’s χ^2^ test. Effect sizes for categorical associations were assessed using Phi coefficients or Cramer’s V, as appropriate.

Pairwise correlations between extracted maternal vulnerability indicators were explored using Kendall’s tau-b correlation analysis and visualized as a heatmap correlation matrix.

Multivariable logistic regression models were constructed to evaluate independent associations between maternal vulnerability indicators and two principal outcomes: documented prenatal care deficiency and low birth weight (<2500 g). To reduce the risk of model overfitting, the number of predictors in the primary models was restricted relative to the number of outcome events, and clinically relevant variables were retained in the final analyses. The prenatal-care-deficiency model included 692 pregnancies, 612 events, and seven predictors, corresponding to 87.4 events per variable. The primary low-birth-weight model included 666 pregnancies, 80 events, and four predictors, corresponding to 20.0 events per variable. The prenatal-care-deficiency model included smoking, maternal anemia, infection during pregnancy, hypertensive disorders, obesity, Rh incompatibility, and residential environment. The primary adjusted model for low birth weight included maternal anemia, hypertensive disorders, maternal age, and residential environment, based on clinical relevance and univariable association patterns. As a sensitivity analysis, the low-birth-weight regression was repeated using a full-entry specification including all seven predefined maternal vulnerability indicators, maternal age, and residential environment. This model included 666 pregnancies, 80 events, and nine predictors, corresponding to 8.9 events per variable, and was therefore interpreted cautiously as an exploratory sensitivity analysis.

Adjusted odds ratios (aORs) with corresponding 95% confidence intervals (95% CI) were reported. Odds ratios greater than 1 indicated increased odds of the outcome, whereas values below 1 indicated decreased odds after adjustment for the remaining covariates. Multicollinearity among predictors was evaluated using variance inflation factors (VIFs).

Model performance was assessed using likelihood ratio tests and pseudo-R^2^ indices (McFadden and Nagelkerke). Statistical significance was defined as a two-sided *p*-value < 0.05.

## 3. Results

### 3.1. Cohort Characteristics and Documentation Completeness

A total of 759 singleton adolescent pregnancies were included in the final cohort. Maternal age ranged from 12 to 17 years, with a median age of 16 years (IQR 15–17). Most participants originated from rural areas (60.9%).

Among the 759 eligible pregnancies, narrative clinical documentation was available for 692 cases (91.2%), whereas 67 records (8.8%) contained no narrative clinical information suitable for extraction of maternal vulnerability indicators. Because the primary objective of the study was to evaluate maternal vulnerability patterns derived from routine clinical documentation, only pregnancies with available narrative observations contributed to analyses involving narrative-derived variables.

Among pregnancies with available narrative observations, documented prenatal care deficiency was identified in 612 cases (88.4%), representing the most frequently recorded vulnerability-related finding. Median gestational age at delivery was 39 weeks (IQR 38–39), while mean birth weight was 2969 ± 466 g.

Low birth weight (<2500 g) was observed in 85 of 759 neonates (11.2%). Gestational age information was available for 716 pregnancies, among which 84 (11.7%) resulted in preterm birth. Apgar score at 5 min was available for 679 neonates, with 32 cases (4.7%) presenting low neonatal adaptation (Apgar score < 7).

Data completeness was high for most structured obstetric and neonatal variables. Missing information ranged from 5.3% for birth weight to 10.5% for Apgar score at 5 min. The availability of narrative clinical observations in more than 90% of pregnancies provided a substantial basis for evaluating maternal vulnerability patterns derived from routine obstetric documentation.

Baseline maternal and neonatal characteristics of the study cohort are summarized in Table 2.

To assess potential bias related to the availability of narrative clinical observations, pregnancies with and without free-text documentation were compared (Table 3). Cases lacking narrative observations differed significantly only with respect to residential environment, being more frequently observed among urban residents (53.7% vs. 37.7%, χ^2^ = 6.58, *p* = 0.010). No significant differences were identified regarding maternal age (*p* = 0.601), low birth weight (9.4% vs. 12.0%, *p* = 0.576), preterm birth (*p* = 0.876), or Apgar score < 7 at 5 min (*p* = 0.323). These findings indicate that the availability of narrative documentation was not significantly associated with the evaluated obstetric and neonatal outcomes, although it differed according to residential environment. The missingness mechanism is unlikely to be completely random and may reflect systematic differences in clinical documentation practices across healthcare settings.

### 3.2. Independent Internal Validation of the Extraction Framework

The category-specific performance of the frozen extraction framework is presented in Table 4. Complete concordance was observed for documented prenatal care deficiency, smoking during pregnancy, maternal anemia, infectious conditions during pregnancy, maternal obesity, and Rh incompatibility. However, the number of clinician-positive cases was limited for infectious conditions (n = 6), maternal obesity (n = 3), and Rh incompatibility (n = 10), resulting in wide confidence intervals despite observed sensitivities of 100%.

For hypertensive disorders, the framework identified four of the seven clinician-positive records, corresponding to a sensitivity of 57.1% (95% CI 18.4–90.1%), an agreement of 97.0%, and Cohen’s κ of 0.713.

In the primary analysis using the clinician-confirmed corrected classifications, 697 of 700 assignments were concordant. Because the three category data-entry errors were identified after comparison of the clinician and rule-based classifications, a sensitivity analysis was performed using the original clinician entries. In this uncorrected analysis, 694 of 700 assignments were concordant (99.1%; descriptive pooled Cohen’s κ = 0.977). Sensitivity was 75.0% for infectious conditions, 57.1% for hypertensive disorders, and 75.0% for maternal obesity.

### 3.3. Distribution of Narrative-Derived Maternal Vulnerability Indicators

The available narrative clinical documentation was systematically reviewed to identify recurrent maternal vulnerability indicators recorded during the hospitalization associated with delivery (Table 5). Among pregnancies with available narrative observations (n = 692), documented prenatal care deficiency was identified in 612 cases (88.4%).

Among the remaining extracted variables, maternal anemia was the most common documented clinical condition, occurring in 207 pregnancies (29.9%), followed by smoking during pregnancy in 186 cases (26.9%). Rh incompatibility and infectious conditions during pregnancy were documented less frequently, affecting 65 (9.4%) and 57 (8.2%) pregnancies, respectively. Hypertensive disorders and maternal obesity were relatively uncommon, being identified in only 19 (2.7%) and 35 (5.1%) pregnancies.

Overall, the distribution of documented maternal vulnerability indicators was highly heterogeneous. While behavioral and healthcare-engagement-related observations such as smoking and documented prenatal care deficiency were relatively frequent, chronic maternal conditions such as obesity and hypertensive disorders were recorded only in a small proportion of cases.

Missingness was consistent across all narrative-derived variables because the absence of narrative documentation affected entire records rather than individual indicators. Consequently, the proportion of missing data was identical (8.8%) for all maternal vulnerability indicators included in the extraction framework.

The substantially higher frequency of documented prenatal care deficiency compared with the other vulnerability indicators indicates that healthcare engagement and prenatal surveillance were the most prominent dimensions captured in the available narrative documentation.

### 3.4. Correlation Structure of Maternal Vulnerability Indicators

**Figure 3:**
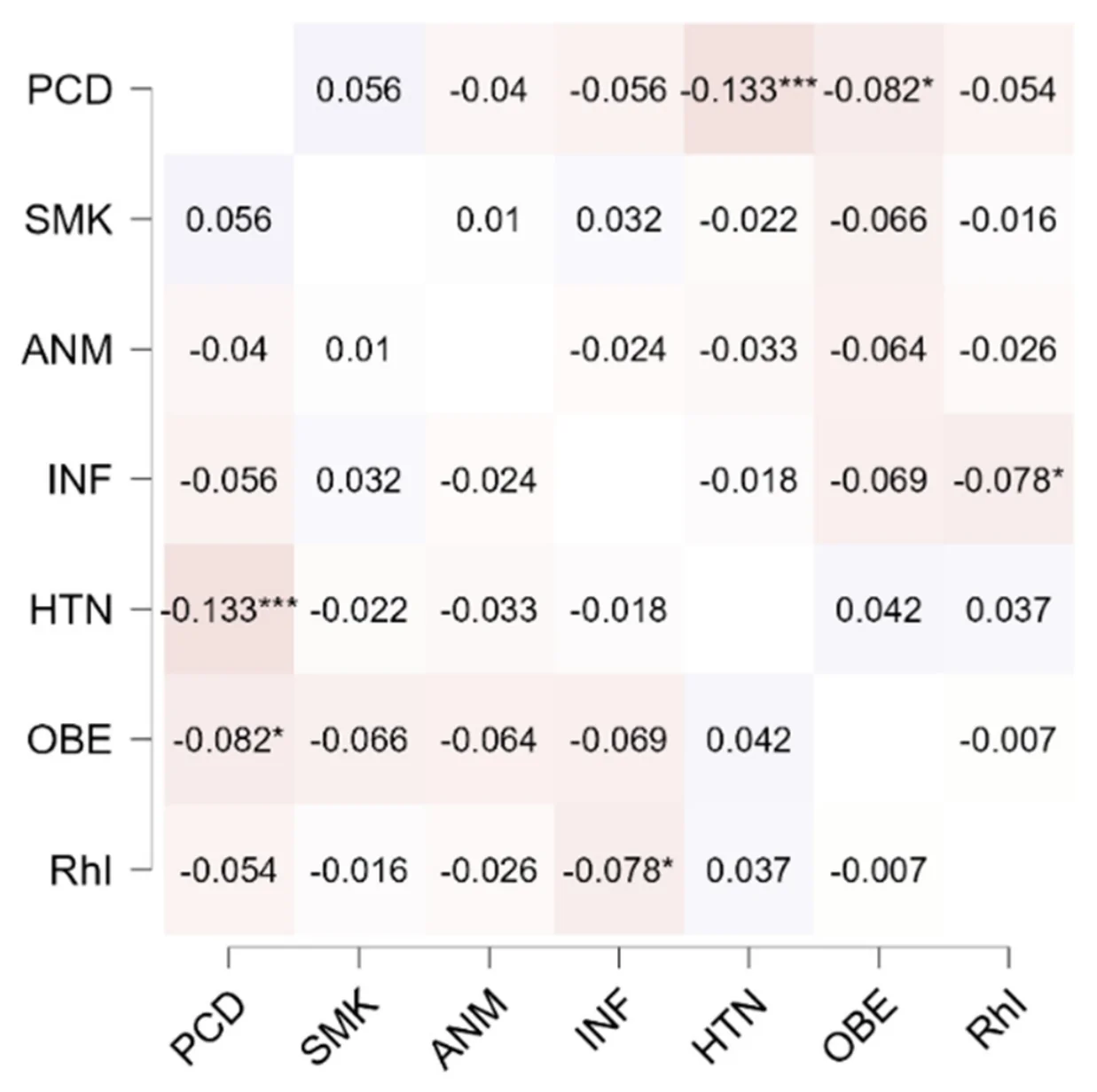
Kendall’s tau-b correlation matrix of narrative-derived maternal vulnerability indicators. Abbreviations: PCD, prenatal care deficiency; SMK, smoking during pregnancy; ANM, maternal anemia; INF, infection during pregnancy; HTN, hypertensive disorders; OBE, maternal obesity; RhI, Rh incompatibility. * *p* < 0.05; *** *p* < 0.001.

Pairwise correlations between narrative-derived maternal vulnerability indicators were generally weak (Figure 3). The strongest associations involved documented prenatal care deficiency and both hypertensive disorders (τ = −0.133, *p* < 0.001) and maternal obesity (τ = −0.082, *p* = 0.032), although both correlations remained weak in magnitude.

Correlations were uniformly weak, with absolute Kendall’s tau-b coefficients ranging from 0.007 to 0.133. Apart from the inverse associations observed between documented prenatal care deficiency and chronic maternal conditions, the correlation matrix revealed only minimal relationships between the evaluated indicators.

Overall, the weak correlation structure suggests that the extracted vulnerability indicators represented relatively independent dimensions of maternal vulnerability rather than a highly clustered risk profile.

### 3.5. Associations Between Maternal Vulnerability Indicators and Documented Prenatal Care Deficiency

Univariable analyses were performed to evaluate the association between narrative-derived maternal vulnerability indicators and documented prenatal care deficiency (Table 6). Significant associations were identified for hypertensive disorders and maternal obesity, although both effect sizes remained small. Hypertensive disorders were documented in 8.8% of pregnancies without documented prenatal care deficiency compared with 2.0% of pregnancies with documented prenatal care deficiency (*p* < 0.001). Similarly, maternal obesity was observed in 10.0% and 4.4% of pregnancies, respectively (*p* = 0.032).

No statistically significant associations were identified for smoking during pregnancy, maternal anemia, infectious conditions during pregnancy, or Rh incompatibility. Effect sizes were small throughout the analysis (Phi range: 0.040–0.133), indicating that although statistically significant associations were observed for hypertensive disorders and obesity, the overall magnitude of these relationships remained limited.

### 3.6. Multivariable Logistic Regression Analysis for Factors Associated with Documented Prenatal Care Deficiency

A multivariable logistic regression model was constructed to identify factors independently associated with documented prenatal care deficiency (Table 7).

The overall model demonstrated a statistically significant improvement over the null model (Δχ^2^ = 20.75, *p* = 0.004), although explanatory performance remained modest (Nagelkerke R^2^ = 0.058).

Hypertensive disorders (aOR 0.31, 95% CI 0.10–0.95, *p* = 0.038) and maternal obesity (aOR 0.39, 95% CI 0.16–0.98, *p* = 0.044) were associated with lower odds of documented prenatal care deficiency and were more frequently documented among pregnancies with recorded prenatal surveillance.

Smoking during pregnancy, maternal anemia, infectious conditions during pregnancy, Rh incompatibility, and residential environment were not independently associated with prenatal care documentation status.

Although statistically significant, the model explained only a limited proportion of the variability in prenatal care documentation status (Nagelkerke R^2^ = 0.058), indicating that additional clinical and social determinants not captured in the available records likely contributed to differences in prenatal surveillance patterns.

### 3.7. Associations Between Maternal Vulnerability Indicators and Neonatal Outcomes

Univariable analyses demonstrated selective associations between narrative-derived maternal vulnerability indicators and neonatal outcomes. Complete results for all seven indicators and all three neonatal outcomes are presented in Table 8.

Significant associations were identified only for low birth weight (LBW). Hypertensive disorders were associated with a higher prevalence of LBW, with low birth weight observed in 31.6% of pregnancies complicated by hypertension compared with 11.4% among pregnancies without hypertensive disorders (*p* = 0.008).

An inverse association was observed for documented maternal anemia. LBW occurred in 6.8% of pregnancies with documented anemia compared with 14.3% among pregnancies without documented anemia (*p* = 0.006). Given the observational nature of the study and the reliance on routinely documented clinical information, this finding should not be interpreted as evidence of a protective effect of anemia. Instead, documented anemia may reflect increased clinical surveillance, diagnostic ascertainment, and therapeutic management during pregnancy.

Documented prenatal care deficiency, smoking during pregnancy, infection during pregnancy, maternal obesity, and Rh incompatibility were not significantly associated with LBW. No significant associations were identified between any of the evaluated maternal vulnerability indicators and either preterm birth or a 5 min Apgar score < 7. Across all comparisons, effect sizes were small (Phi ≤ 0.107), indicating limited associations between the narrative-derived indicators and neonatal outcomes.

### 3.8. Multivariable Logistic Regression Analysis for Low Birth Weight

A multivariable logistic regression model was constructed to evaluate the independent association between selected maternal vulnerability indicators and low birth weight (Table 9). The model included 666 pregnancies, of which 80 had low birth weight, and four predictors, corresponding to 20.0 events per variable. The overall model demonstrated a statistically significant improvement compared with the null model (Δχ^2^ = 14.99, *p* = 0.005), although explanatory performance remained modest (Nagelkerke R^2^ = 0.043), indicating that only a limited proportion of variability in low birth weight was explained by the variables included in the model.

Hypertensive disorders were independently associated with increased odds of low birth weight (aOR 3.56, 95% CI 1.33–9.54, *p* = 0.011). In contrast, documented maternal anemia was associated with lower observed odds of LBW (aOR 0.45, 95% CI 0.24–0.82, *p* = 0.009). Maternal age and residential environment were not significantly associated with the outcome after adjustment.

Although statistically significant, the inverse association observed for documented maternal anemia should not be interpreted as evidence of a protective biological effect. Given that anemia was identified through routine clinical documentation, its presence may reflect increased clinical surveillance, diagnostic ascertainment, and subsequent therapeutic management during pregnancy.

To assess whether these findings were dependent on the predictor-selection strategy, the low-birth-weight regression was repeated using a full-entry specification that included all seven predefined maternal vulnerability indicators, maternal age, and residential environment. This model included 666 pregnancies, 80 low-birth-weight events, and nine predictors, corresponding to 8.9 events per variable. The sensitivity model was statistically significant (Δχ^2^ = 19.49, *p* = 0.021; Nagelkerke R^2^ = 0.055). The principal associations were directionally consistent with those observed in the primary model: documented maternal anemia was associated with lower observed odds of low birth weight (aOR 0.43, 95% CI 0.24–0.80, *p* = 0.007), whereas hypertensive disorders were associated with increased odds of low birth weight (aOR 3.21, 95% CI 1.13–9.17, *p* = 0.029). The remaining vulnerability indicators, maternal age, and residential environment were not independently associated with low birth weight. Multicollinearity was negligible, with all variance inflation factors below 1.11. Because the full-entry model had an events-per-variable ratio below 10, its estimates should be interpreted cautiously. The complete results are presented in Table 10.

## 4. Discussion

### 4.1. Principal Findings

This study characterized selected maternal vulnerability indicators derived from narrative clinical documentation and examined their exploratory associations with prenatal surveillance patterns and neonatal outcomes. The present analysis complements two previous studies based on the same institutional cohort, which evaluated age-related obstetric patterns and parity-related neonatal outcomes using structured clinical variables [20,21]. In contrast, the current study focused specifically on maternal vulnerability indicators extracted from routine obstetric narratives that had no corresponding structured variables in the available analytic dataset. By transforming these narrative observations into predefined epidemiological variables, the study examined their distribution, interrelationships, and exploratory associations with documented prenatal care deficiency and neonatal outcomes.

Overall, the findings revealed a heterogeneous vulnerability profile in which documented maternal risk indicators showed limited clustering and weak interrelationships. Even when statistically significant, the observed associations were generally characterized by small effect sizes, indicating limited practical strength of the relationships. Within this framework, documented prenatal care deficiency emerged as the dominant vulnerability-related observation, emphasizing the importance of healthcare engagement and documentation processes in shaping the information captured in routine obstetric records. At the same time, chronic maternal conditions such as hypertensive disorders and obesity were more frequently documented among pregnancies receiving antenatal surveillance, suggesting that free-text observations reflect not only underlying morbidity but also opportunities for clinical detection and documentation. The relationship between narrative-derived vulnerability indicators and neonatal outcomes was similarly selective, with significant associations observed primarily for low birth weight rather than for prematurity or impaired neonatal adaptation.

Taken together, these findings suggest that the extracted narrative indicators represented selected dimensions of maternal vulnerability while also reflecting healthcare utilization and documentation practices. Rather than representing a unified high-risk profile, narrative-derived indicators appear to reflect multiple and partially independent dimensions of vulnerability that together shape the clinical picture recorded during routine obstetric care.

### 4.2. Documented Prenatal Care Deficiency and Healthcare Engagement

The analysis revealed a substantial burden of documented prenatal care deficiency captured through routine clinical documentation. Among pregnancies with available narrative observations, nearly 90% contained clinician-recorded indications of absent, delayed, irregular, or insufficient antenatal surveillance. Importantly, this proportion should not be interpreted as a population-level estimate of inadequate antenatal care among all adolescent pregnancies. Rather, it reflects clinician-documented observations recorded within a secondary referral cohort and is likely influenced by referral patterns, healthcare utilization, and documentation practices.

The high prevalence of documented prenatal care deficiency is nevertheless consistent with previous research showing that pregnant adolescents frequently encounter barriers to antenatal care utilization. Socioeconomic disadvantage, limited autonomy, delayed pregnancy disclosure, financial constraints, and reduced access to adolescent-friendly healthcare services have all been associated with delayed initiation and reduced continuity of prenatal care among adolescent mothers [22,23,24,25]. These challenges often coexist with broader social vulnerabilities, including educational disadvantage, unintended pregnancy, and reduced access to reproductive health resources [26,27].

However, documented prenatal care deficiency was not independently associated with adverse neonatal outcomes in the present study. This finding suggests that clinician-recorded deficiencies in prenatal surveillance may not function as direct markers of neonatal risk when extracted from routine narrative documentation. Instead, they may reflect documentation patterns influenced by healthcare utilization, organizational factors, and clinical context. Because free-text observations capture clinician-recorded assessments rather than standardized measures of antenatal care adequacy, documented prenatal care deficiency should be interpreted as an indicator situated at the intersection of maternal vulnerability, healthcare access, and clinical documentation practices.

### 4.3. Heterogeneity of Maternal Vulnerability Indicators

An important finding of the present study was the highly heterogeneous structure of the extracted maternal vulnerability indicators. Pairwise correlations were consistently weak, with most Kendall’s tau-b coefficients remaining below 0.10, and no clear clustering pattern emerged among the evaluated variables. Although several correlations reached statistical significance, their magnitude remained small, suggesting limited practical interdependence among the evaluated indicators. Therefore, statistical significance should not be interpreted as evidence of strong clinical relationships between the different dimensions of vulnerability.

These findings suggest that narrative-derived maternal vulnerability indicators do not represent a unified high-risk phenotype. Instead, they appear to capture multiple, partially independent dimensions of vulnerability that encompass biological, behavioral, and healthcare-related characteristics. Such fragmentation is not unexpected in adolescent pregnancy, where vulnerability arises through the interaction of medical, social, developmental, and healthcare-access factors rather than through a single dominant risk pathway.

The observed heterogeneity may also reflect characteristics of routine clinical documentation. Narrative observations are generated within specific healthcare encounters and are influenced by diagnostic opportunities, clinician priorities, documentation practices, and healthcare utilization patterns [28,29,30]. Consequently, free-text-derived indicators may simultaneously reflect biological vulnerability and healthcare-system interactions. This multidimensional structure may explain the limited clustering observed between indicators and their selective associations with neonatal outcomes.

### 4.4. Documentation Intensity, Clinical Detection, and Maternal Vulnerability

One notable finding of the present study was the consistent association between chronic maternal conditions and prenatal surveillance patterns. Both hypertensive disorders and maternal obesity were more frequently documented among pregnancies without documented prenatal care deficiency and remained independently associated with antenatal surveillance in multivariable analyses.

These findings are unlikely to indicate that inadequate prenatal surveillance protects against chronic maternal conditions. Rather, they support the interpretation that clinically detectable conditions are more likely to be identified, investigated, and documented among pregnancies receiving regular healthcare follow-up. In this context, the presence of a documented diagnosis may reflect not only underlying disease but also opportunities for clinical detection.

This observation is consistent with the concept of informed presence bias, whereby patients with more frequent healthcare encounters accumulate a greater volume of documented clinical information simply because they interact more often with healthcare services [10]. As a result, the apparent prevalence of some conditions may be influenced by differences in healthcare utilization and diagnostic intensity rather than by true epidemiological differences. Similarly, the higher proportion of pregnancies lacking narrative observations among urban residents may reflect variation in documentation practices, healthcare workflows, referral pathways, or patterns of healthcare engagement. However, the available data do not permit direct evaluation of these mechanisms, and this interpretation should therefore be considered speculative.

Recent research has emphasized that clinical documentation is influenced by healthcare encounters, clinician workflows, and documentation practices rather than representing a purely objective record of patient status [28,29,30]. Consequently, variables derived from narrative documentation may simultaneously reflect disease burden, diagnostic opportunity, healthcare utilization, and documentation practices.

The present findings illustrate this phenomenon in adolescent pregnancy, where conditions such as hypertension and obesity require clinical recognition and documentation before becoming visible within electronic health records. Therefore, pregnancies receiving more regular antenatal surveillance provide greater opportunities for detection and recording of these conditions. Accordingly, the observed associations are best interpreted as indicators of clinical detection and documentation opportunity rather than evidence of biological protection conferred by prenatal surveillance patterns.

### 4.5. Maternal Vulnerability Indicators and Neonatal Outcomes

The associations between narrative-derived maternal vulnerability indicators and neonatal outcomes were selective rather than generalized. Significant relationships were identified primarily for low birth weight, whereas preterm birth and low Apgar score at 5 min showed no meaningful associations with the evaluated indicators. This pattern suggests that the information captured within routine clinical documentation may reflect specific dimensions of fetal growth vulnerability more effectively than broader neonatal risk.

Hypertensive disorders emerged as the strongest neonatal risk factor identified in the present study, remaining independently associated with low birth weight in multivariable analyses. This finding is biologically plausible and consistent with extensive evidence linking maternal hypertensive disorders to placental dysfunction, impaired uteroplacental perfusion, fetal growth restriction, and reduced birth weight. Recent prospective and population-based studies continue to identify hypertensive disorders among the most important maternal predictors of fetal growth impairment and adverse perinatal outcomes [15].

In contrast, the evaluated vulnerability indicators demonstrated little association with preterm birth. This observation is noteworthy because prematurity is widely recognized as a multifactorial outcome influenced by a complex interplay of maternal, fetal, placental, obstetric, behavioral, and socioeconomic determinants. Recent umbrella reviews have highlighted the broad spectrum of factors contributing to preterm birth, many of which were not available within the present dataset [17]. Consequently, the absence of significant associations may reflect the limited ability of routine narrative observations to capture the full range of pathways leading to premature delivery.

Similarly, no meaningful associations were observed between the extracted vulnerability indicators and neonatal adaptation as measured by the Apgar score at 5 min. This finding is consistent with evidence indicating that Apgar scores are influenced by multiple factors, including gestational maturity, intrapartum events, delivery-related complications, and the newborn’s immediate clinical condition [18]. The relationship between Apgar scores and umbilical cord blood acid–base balance is also imperfect, and intrapartum conditions such as clinically significant nuchal cord entanglement may further affect immediate neonatal adaptation [31,32].

### 4.6. The Paradoxical Association Between Documented Maternal Anemia and Low Birth Weight

The inverse association between documented maternal anemia and low birth weight was biologically counterintuitive, as maternal anemia has consistently been identified as a risk factor for adverse fetal growth outcomes [33,34,35].

Several explanations may account for this apparent paradox. First, the present study relied on routine clinical documentation rather than standardized prospective assessment of maternal anemia. Consequently, the variable analyzed reflects documented anemia rather than the true prevalence of anemia within the cohort. Pregnancies in which anemia was identified and recorded necessarily involved healthcare contact, laboratory testing, clinical recognition, and, in many cases, therapeutic intervention. Documentation therefore likely represents a marker of diagnostic ascertainment rather than a simple indicator of disease presence [10,28,29,30].

Second, recognition of anemia may have triggered closer clinical monitoring, nutritional counseling, iron supplementation, or additional follow-up during pregnancy. Under such circumstances, documented anemia could paradoxically become associated with improved management and partial mitigation of fetal growth risks. Similar mechanisms have been described in observational studies based on electronic health records, where documented conditions may reflect diagnostic opportunity, clinical surveillance, and documentation practices rather than disease severity alone [10,28,29,30].

Third, residual confounding and reverse causation cannot be excluded. Important determinants of both maternal anemia and fetal growth, including nutritional status, socioeconomic conditions, dietary quality, timing of antenatal care initiation, treatment adherence, and anemia severity, were unavailable within the present dataset. Accordingly, despite statistical significance, the observed association should be interpreted cautiously and not considered evidence of a protective biological effect.

Taken together, these findings reinforce a broader methodological message emerging from the present study: variables derived from routine narrative documentation may simultaneously capture biological conditions, diagnostic opportunity, clinical surveillance, documentation practices, and therapeutic management. Interpretation of such variables therefore requires careful consideration of the clinical context in which documentation is generated.

### 4.7. Strengths and Limitations

The present study possesses several methodological strengths. The analysis was conducted in a relatively large cohort of adolescent pregnancies managed within a single secondary referral institution using a consistent clinical documentation system. Narrative clinical observations were available for the vast majority of eligible pregnancies, providing a substantial basis for the evaluation of free-text-derived maternal vulnerability indicators. In addition, the study combined systematic free-text harmonization with clinically guided variable extraction, enabling the incorporation of contextual information that is often unavailable within structured hospital databases. By integrating correlation analyses, healthcare-engagement models, and neonatal outcome assessments, the study offers a multidimensional perspective on maternal vulnerability captured through routine clinical documentation.

Several limitations should be acknowledged. The retrospective, single-center design precludes causal inference and may limit the generalizability of the findings to other healthcare settings. Narrative extraction relied on clinician-defined harmonization and rule-based categorization rather than advanced natural language processing methods, which may have resulted in underdetection or misclassification of some clinical conditions. Furthermore, the extracted variables represented selected vulnerability-related indicators identifiable within the available routine clinical documentation rather than a comprehensive assessment of maternal vulnerability. Important psychological, social, economic, and environmental determinants, including socioeconomic status, educational level, nutritional factors, family support, timing of antenatal care initiation, and treatment adherence, were not available in the source documentation and could not be incorporated into the analyses.

A further limitation relates to the data-generation process inherent to routine clinical documentation. The recorded information reflects not only patient characteristics but also healthcare utilization, diagnostic opportunity, clinician priorities, referral pathways, and documentation practices. Differences in documentation intensity and healthcare engagement may therefore influence the apparent prevalence and distribution of narrative-derived variables, introducing information and ascertainment biases that cannot be fully eliminated in retrospective research based on routinely collected health data [10]. These methodological challenges are consistent with broader concerns regarding the interpretation of routinely generated clinical data and are recognized in reporting frameworks such as the RECORD initiative [19].

The structured component of the analytic dataset did not include systematically coded variables corresponding to the seven narrative-derived maternal vulnerability indicators. Consequently, the study could not quantify agreement between structured and unstructured documentation, determine the number of cases identified exclusively through narrative review, assess changes in patient classification after the addition of narrative information, or estimate the incremental informational or prognostic value of the narrative-derived indicators beyond the available structured variables. Future studies linking structured diagnoses, laboratory findings, and narrative documentation are required to evaluate these dimensions directly.

The relatively low frequency of some conditions, particularly hypertensive disorders and maternal obesity, may also have reduced statistical precision and contributed to wide confidence intervals for some regression estimates. Accordingly, non-significant findings should not necessarily be interpreted as evidence of no association. The principal low-birth-weight associations were directionally consistent in the full-entry sensitivity analysis. However, this model included 80 events and nine predictors, corresponding to 8.9 events per variable; its estimates may therefore be unstable and should be interpreted cautiously. Alternative analytical approaches, including penalized regression, were not assessed.

Pregnancies without available narrative documentation were broadly comparable with those with narrative information regarding major maternal and neonatal characteristics, which reduces concern about substantial outcome-related selection bias. Nevertheless, residual selection bias cannot be excluded, because the availability and content of narrative documentation may also reflect unmeasured differences in clinical complexity, healthcare utilization, referral patterns, or documentation practices.

Although the frozen extraction framework demonstrated high agreement in an independent internal validation sample, validation was performed within the same institutional documentation environment. External validation in another hospital, documentation system, or patient population was not performed. Moreover, agreement was lower for hypertensive disorders than for the other categories, indicating greater interpretative variability for this indicator. The internal validation assessed the reproducibility of category assignment but did not independently establish the clinical validity or prognostic utility of the extracted categories. Three clinician classification entries were corrected after comparison with the rule-based classifications; therefore, validation results are reported both using the clinician-confirmed corrected entries and retaining the original clinician classifications.

The validation sample contained few positive cases for several uncommon indicators, particularly maternal obesity and infectious conditions. Consequently, estimates of sensitivity and positive predictive value for these categories had wide confidence intervals and should not be interpreted as precise evidence of perfect classification performance. For hypertensive disorders, sensitivity was lower and imprecisely estimated, indicating that the rule-based framework may fail to identify some cases classified as positive by the independent clinician. Larger validation samples enriched for rare positive categories are needed to estimate category-specific sensitivity more reliably.

Taken together, these limitations indicate that the findings should be regarded as exploratory. Their interpretation should account for the weak effect sizes, limited explanatory performance of the regression models, absence of direct comparison with corresponding structured data, and lack of external validation.

### 4.8. Clinical and Research Implications

The present findings illustrate how selected maternal vulnerability indicators can be identified in routinely collected narrative clinical documentation, while also underscoring the need to consider healthcare engagement, diagnostic opportunity, and documentation practices when interpreting such variables. The extracted indicators should therefore be regarded as documentation-based measures rather than direct or comprehensive representations of underlying maternal vulnerability.

Future studies should integrate narrative documentation with structured diagnoses, laboratory findings, longitudinal prenatal-care data, and relevant social determinants of health. Direct comparisons between structured and unstructured data sources are needed to quantify additional case detection, concordance, patient reclassification, and incremental prognostic contribution. External validation in other institutions and documentation systems is also required to determine the generalizability of the extraction framework. Advanced natural language processing approaches may facilitate broader-scale extraction, but their performance should be evaluated against clinically defined reference standards and interpreted within the healthcare context in which the documentation was generated.

Until such evidence is available, the principal contribution of the present study is methodological and exploratory: it demonstrates a reproducible approach for structuring selected information from routine obstetric narratives while highlighting the interpretative constraints inherent to documentation-derived variables.

## 5. Conclusions

Free-text clinical documentation allowed the identification of heterogeneous maternal vulnerability indicators in this cohort of adolescent pregnancies. The extracted indicators showed weak interrelationships and selective associations with prenatal surveillance patterns and low birth weight, while no significant associations were observed for preterm birth or low Apgar score at 5 min.

The findings also suggest that narrative-derived variables may reflect not only maternal clinical characteristics but also healthcare engagement, diagnostic opportunity, and documentation practices. Accordingly, these variables should be interpreted cautiously and within the context in which the clinical documentation was generated.

Because the study did not compare narrative-derived indicators with corresponding structured diagnoses or codes, did not assess incremental prognostic value, and was conducted in a single-center retrospective cohort, the results should be regarded as exploratory. Further studies using external validation cohorts and linked structured and unstructured clinical data are required to determine the reproducibility, incremental informational value, and clinical utility of narrative-derived maternal vulnerability indicators.

## Figures and Tables

**Table 1:** Harmonization dictionary used for narrative-data extraction and category assignment.

Maternal Vulnerability Category	Standardized Narrative Expressions Included in the Category
Documented prenatal care deficiency	insufficiently investigated pregnancy, uninvestigated pregnancy, non-dispensarized pregnancy
Smoking during pregnancy	smoker, smoking, tobacco use
Maternal anemia	anemia, secondary anemia, iron-deficiency anemia
Infectious conditions during pregnancy	urinary tract infection, urinary infection during pregnancy, *Escherichia coli* urinary infection, bladder infection, vulvovaginitis, genital tract infection, gonococcal infection, respiratory infection, acute respiratory infection
Hypertensive disorders	gestational hypertension, pregnancy-induced hypertension, hypertension during pregnancy, preeclampsia, eclampsia
Maternal obesity	obesity, grade II obesity, morbid obesity, pyknic habitus, obesity acquired during pregnancy
Rh incompatibility	Rh-negative pregnancy, Rh isoimmunization, Rh incompatibility, Rh-negative mother with Rh-positive newborn, anti-D immunoglobulin administration related to Rh incompatibility

The listed expressions represent standardized terms generated from multiple synonymous or orthographically variable narrative formulations identified in the original clinical documentation.

**Table 2:** Baseline maternal and neonatal characteristics of the study cohort (n = 759).

Variable	Value
Maternal age, median (IQR)	16 (15–17) years
Rural residence, n (%)	462 (60.9%)
Documented prenatal care deficiency, n/N (%)	612/692 (88.4%)
Gestational age, median (IQR)	39 (38–39) weeks
Birth weight, mean ± SD	2969 ± 466 g
Low birth weight (<2500 g), n (%)	85 (11.2%)
Preterm birth (<37 weeks), n/N (%)	84/716 (11.7%)
Apgar score < 7 at 5 min, n/N (%)	32/679 (4.7%)
Narrative documentation available, n (%)	692 (91.2%)
No narrative documentation, n (%)	67 (8.8%)

**Table 3:** Comparison of pregnancies with and without available free-text observations.

Variable	No Narrative Documentation (n = 67)	Narrative Documentation Available (n = 692)	*p*
Maternal age, mean ± SD	16.06 ± 1.01	15.97 ± 1.08	0.601
Urban residence, n (%)	36 (53.7%)	261 (37.7%)	0.010
Low birth weight, n/N (%)	5/53 (9.4)	80/666 (12.0)	0.576
Preterm birth, n/N (%)	7/54 (13.0)	81/662 (12.2)	0.876
Apgar < 7 at 5 min, n/N (%)	1/52 (1.9)	31/627 (4.9)	0.323

**Table 4:** Category-specific performance of the frozen rule-based extraction framework in the independent validation sample.

Indicator	TP	FN	FP	TN	Sensitivity, % (95% CI)	Specificity, % (95% CI)	PPV, % (95% CI)	NPV, % (95% CI)	Agreement, %	Cohen’s κ
Primary analysis using the clinician-confirmed corrected classifications										
Documented prenatal care deficiency	88	0	0	12	100 (95.9–100)	100 (73.5–100)	100 (95.9–100)	100 (73.5–100)	100	1.000
Smoking during pregnancy	30	0	0	70	100 (88.4–100)	100 (94.9–100)	100 (88.4–100)	100 (94.9–100)	100	1.000
Maternal anemia	32	0	0	68	100 (89.1–100)	100 (94.7–100)	100 (89.1–100)	100 (94.7–100)	100	1.000
Infectious conditions during pregnancy	6	0	0	94	100 (54.1–100)	100 (96.2–100)	100 (54.1–100)	100 (96.2–100)	100	1.000
Hypertensive disorders	4	3	0	93	57.1 (18.4–90.1)	100 (96.1–100)	100 (39.8–100)	96.9 (91.1–99.4)	97.0	0.713
Maternal obesity	3	0	0	97	100 (29.2–100)	100 (96.3–100)	100 (29.2–100)	100 (96.3–100)	100	1.000
Rh incompatibility	10	0	0	90	100 (69.2–100)	100 (96.0–100)	100 (69.2–100)	100 (96.0–100)	100	1.000
Sensitivity analysis using the original, uncorrected clinician classifications										
Infectious conditions during pregnancy	6	2	0	92	75.0 (34.9–96.8)	100 (96.1–100)	100 (54.1–100)	97.9 (92.5–99.7)	98.0	0.847
Hypertensive disorders	4	3	0	93	57.1 (18.4–90.1)	100 (96.1–100)	100 (39.8–100)	96.9 (91.1–99.4)	97.0	0.713
Maternal obesity	3	1	0	96	75.0 (19.4–99.4)	100 (96.2–100)	100 (29.2–100)	99.0 (94.4–100)	99.0	0.852

TP, true positive; FN, false negative; FP, false positive; TN, true negative; PPV, positive predictive value; NPV, negative predictive value. The sensitivity analysis reports only the categories affected by the original clinician data-entry errors; results for the remaining categories were identical to those in the primary analysis.

**Table 5:** Distribution of narrative-derived maternal vulnerability indicators among pregnancies with available narrative documentation (N = 692).

Variable	Present n (%)	Absent n (%)
Documented prenatal care deficiency	612 (88.4)	80 (11.6)
Smoking during pregnancy	186 (26.9)	506 (73.1)
Maternal anemia	207 (29.9)	485 (70.1)
Infection during pregnancy	57 (8.2)	635 (91.8)
Hypertensive disorders	19 (2.7)	673 (97.3)
Maternal obesity	35 (5.1)	657 (94.9)
Rh incompatibility	65 (9.4)	627 (90.6)

Percentages are based on the 692 pregnancies with available narrative documentation. The 67 pregnancies without narrative observations were not classifiable for these indicators and were excluded from the denominator.

**Table 6:** Associations between maternal vulnerability indicators and documented prenatal care deficiency.

Variable	No Documented Prenatal Care Deficiency n/N (%)	Documented Prenatal Care Deficiency n/N (%)	*p*-Value	Phi
Smoking	16/80 (20.0%)	170/612 (27.8%)	0.140	0.056
Anemia	28/80 (35.0%)	179/612 (29.2%)	0.291	0.040
Infection	10/80 (12.5%)	47/612 (7.7%)	0.140	0.056
Hypertensive disorders	7/80 (8.8%)	12/612 (2.0%)	<0.001	0.133
Obesity	8/80 (10.0%)	27/612 (4.4%)	0.032	0.082
Rh incompatibility	11/80 (13.8%)	54/612 (8.8%)	0.155	0.054

**Table 7:** Multivariable logistic regression analysis for factors associated with documented prenatal care deficiency.

Variable	aOR	95% CI	*p*-Value
Smoking during pregnancy	1.47	0.82–2.66	0.197
Maternal anemia	0.70	0.42–1.16	0.168
Hypertensive disorders	0.31	0.10–0.95	0.038
Infection during pregnancy	0.48	0.22–1.02	0.057
Maternal obesity	0.39	0.16–0.98	0.044
Rh incompatibility	0.58	0.27–1.22	0.152
Urban residence	0.82	0.50–1.33	0.420

Outcome: documented prenatal care deficiency (reference category = no documented prenatal care deficiency).

**Table 8:** Univariable associations between maternal vulnerability indicators and neonatal outcomes.

Indicator	Outcome Among Indicator-Present Pregnancies, n/N (%)	Outcome Among Indicator-Absent Pregnancies, n/N (%)	*p*-Value	Phi
Low birth weight (<2500 g); N = 666				
Documented prenatal care deficiency	68/597 (11.4%)	12/69 (17.4%)	0.147	0.056
Smoking during pregnancy	20/179 (11.2%)	60/487 (12.3%)	0.686	0.016
Maternal anemia	14/206 (6.8%)	66/460 (14.3%)	0.006	0.107
Infection during pregnancy	10/54 (18.5%)	70/612 (11.4%)	0.125	0.059
Hypertensive disorders	6/19 (31.6%)	74/647 (11.4%)	0.008	0.103
Maternal obesity	4/34 (11.8%)	76/632 (12.0%)	0.964	0.002
Rh incompatibility	6/64 (9.4%)	74/602 (12.3%)	0.495	0.026
Preterm birth (<37 completed weeks); N = 662				
Documented prenatal care deficiency	75/594 (12.6%)	6/68 (8.8%)	0.365	0.035
Smoking during pregnancy	25/180 (13.9%)	56/482 (11.6%)	0.428	0.031
Maternal anemia	22/206 (10.7%)	59/456 (12.9%)	0.412	0.032
Infection during pregnancy	6/53 (11.3%)	75/609 (12.3%)	0.832	0.008
Hypertensive disorders	2/19 (10.5%)	79/643 (12.3%)	0.818	0.009
Maternal obesity	4/34 (11.8%)	77/628 (12.3%)	0.931	0.003
Rh incompatibility	8/64 (12.5%)	73/598 (12.2%)	0.946	0.003
Apgar score < 7 at 5 min; N = 627				
Documented prenatal care deficiency	29/561 (5.2%)	2/66 (3.0%)	0.448	0.030
Smoking during pregnancy	10/172 (5.8%)	21/455 (4.6%)	0.537	0.025
Maternal anemia	7/193 (3.6%)	24/434 (5.5%)	0.310	0.041
Infection during pregnancy	1/50 (2.0%)	30/577 (5.2%)	0.317	0.040
Hypertensive disorders	2/19 (10.5%)	29/608 (4.8%)	0.254	0.046
Maternal obesity	3/31 (9.7%)	28/596 (4.7%)	0.212	0.050
Rh incompatibility	2/60 (3.3%)	29/567 (5.1%)	0.545	0.024

Percentages were calculated within the indicator-present and indicator-absent groups. Asso-ciations were evaluated using Pearson’s χ^2^ test. Phi coefficients are reported as absolute effect-size values. Analyses were restricted to pregnancies with available narrative documen-tation and complete data for the respective outcome.

**Table 9:** Multivariable logistic regression analysis for low birth weight.

Variable	aOR	95% CI	*p*-Value
Maternal anemia	0.45	0.24–0.82	0.009
Hypertensive disorders	3.56	1.33–9.54	0.011
Maternal age	0.90	0.74–1.11	0.332
Urban residence	0.78	0.48–1.28	0.325

**Table 10:** Full-entry logistic regression sensitivity analysis for low birth weight.

Variable	aOR	95% CI	*p*-Value
Documented prenatal care deficiency	0.60	0.29–1.25	0.171
Smoking during pregnancy	0.93	0.54–1.62	0.810
Maternal anemia	0.43	0.24–0.80	0.007
Infection during pregnancy	1.63	0.77–3.47	0.203
Hypertensive disorders	3.21	1.13–9.17	0.029
Maternal obesity	0.81	0.27–2.42	0.700
Urban residence	0.78	0.47–1.29	0.330
Rh incompatibility	0.68	0.28–1.66	0.394
Maternal age	0.90	0.72–1.11	0.317

Outcome: low birth weight (<2500 g). All seven predefined maternal vulnerability indicators, maternal age, and residential environment were entered simultaneously, without selection based on univariable statistical significance. aOR, adjusted odds ratio; CI, confidence interval.

## Data Availability

The data underlying this article are not publicly available due to institutional and patient confidentiality restrictions but may be made available from the corresponding authors upon reasonable request and with permission from the Arad County Emergency Clinical Hospital.

## Supplementary Materials

The following supporting information can be downloaded at: https://www.mdpi.com/article/10.3390/medicina62081598/s1, Table S1: Original narrative expressions, harmonized terms, and final category assignment.

## Abbreviations

The following abbreviations are used in this manuscript:

ANC	Antenatal Care
ANM	Maternal Anemia
aOR	Adjusted Odds Ratio
CI	Confidence Interval
EHR	Electronic Health Record
HTN	Hypertensive Disorders
INF	Infection During Pregnancy
LBW	Low Birth Weight (<2500 g)
NLP	Natural Language Processing
OBE	Maternal Obesity
OR	Odds Ratio
PCD	Prenatal Care Deficiency
RHI	Rh Incompatibility
SMK	Smoking During Pregnancy
τ	Kendall’s Tau-b Correlation Coefficient
