# Supplementary material for: Maternal Vulnerability Indicators Derived from Free-Text Clinical Documentation in Adolescent Pregnancy
Source: Medicina (Kaunas). 2026 Aug 20;62(8):1598. doi: 10.3390/medicina62081598 (PMC13515706; doi:10.3390/medicina62081598)
Supplement: Supplementary file 1 [file medicina-62-01598-s001.zip › medicina-4473458-supplementary.pdf]

Supplementary Table S1. Original narrative expressions, harmonized terms, and final category assignment

Original narrative expression (Romanian)	Harmonized English term	Final category
sarcină insuficient investigată	insufficiently investigated pregnancy	Documented prenatal care deficiency
sarcină neinvestigată	uninvestigated pregnancy	Documented prenatal care deficiency
sarcină nedispensarizată	non-dispensarized pregnancy	Documented prenatal care deficiency
fumătoare	smoker	Smoking during pregnancy
fumat	smoking	Smoking during pregnancy
consum de tutun	tobacco use	Smoking during pregnancy
anemie	anemia	Maternal anemia
anemie secundară	secondary anemia	Maternal anemia
anemie feriprivă	iron-deficiency anemia	Maternal anemia
infecție tract urinar	urinary tract infection	Infectious conditions during pregnancy
infecție urinară în sarcină	urinary infection during pregnancy	Infectious conditions during pregnancy
infecția vezicii urinare	bladder infection	Infectious conditions during pregnancy
vulvovaginită	vulvovaginitis	Infectious conditions during pregnancy
infecție genitală	genital tract infection	Infectious conditions during pregnancy
infecție gonococică	gonococcal infection	Infectious conditions during pregnancy
infecție respiratorie	respiratory infection	Infectious conditions during pregnancy
IACRS	acute respiratory infection	Infectious conditions during pregnancy
infecție urinară cu *Escherichia coli*	*Escherichia coli* urinary infection	Infectious conditions during pregnancy
hipertensiune gestațională	gestational hypertension	Hypertensive disorders
HTA indusă de sarcină	pregnancy-induced hypertension	Hypertensive disorders
hipertensiune în sarcină	hypertension during pregnancy	Hypertensive disorders
preeclampsie	preeclampsia	Hypertensive disorders
eclampsie	eclampsia	Hypertensive disorders
obezitate	obesity	Maternal obesity

obezitate grad II	grade II obesity	Maternal obesity
obezitate dobândită în sarcină	obesity acquired during pregnancy	Maternal obesity
obezitate morbidă	morbid obesity	Maternal obesity
habitus picnic	pyknic habitus	Maternal obesity
Picnică	pyknic habitus	Maternal obesity
Rh negativ	Rh-negative pregnancy	Rh incompatibility
Rh negativ cu nou-născut Rh pozitiv	Rh-negative mother with Rh-positive newborn	Rh incompatibility
incompatibilitate Rh	Rh incompatibility	Rh incompatibility
izoimunizare Rh	Rh isoimmunization	Rh incompatibility
administrare imunoglobulină anti-D	anti-D immunoglobulin administration related to Rh incompatibility	Rh incompatibility

Original Romanian narrative expressions identified in routine clinical records were translated, harmonized, and assigned to predefined maternal vulnerability categories prior to statistical analysis.
